# At a crossroads: HIV response in southeast Asia

**DOI:** 10.1016/j.lansea.2025.100671

**Published:** 2025-09-10

**Authors:** The Lancet Regional Health – Southeast Asia

Although 42 years have passed since the identification of a new virus—lymphadenopathy-associated virus, or as it is better known today, HIV—it is possible to grasp the profound alarm and uncertainty that accompanied its emergence. In the early 1980s, a diagnosis of HIV was shrouded in stigma and akin to a death sentence. Public discourse at the time was steeped in fear and misinformation, provoking the othering of those affected. Within southeast Asia, as in much of the world, diagnostic facilities and treatment options were severely limited, which only served to compound the marginalisation of those living with the virus. While stigma continues to strongly persist in the region, the overall trajectory over the past 40 years has been one of progress, though it remains to be seen whether the achievements can be sustained and built upon in the wake of ongoing challenges.

Much of the progress to date can be attributed to the advent of safe and effective antiretroviral therapy (ART), which has transformed HIV from a dreaded diagnosis into a chronic, manageable condition. This advancement is reflected in the global decline of both new infections and HIV-related deaths. Recent advances, such as the long-acting antiretroviral medication lenacapavir, which can prevent HIV with twice-yearly injections, are a cause for renewed hope. Similar progress has been recorded regionally. Recent data from WHO show that since 2010, deaths associated with HIV have fallen by 77% in the region and new infections have dropped to fewer than 0.1 per 1000 uninfected people. ART, once scarce, is now available in every country in the WHO South-East Asia region for people living with HIV. In India, for example, ART is provided through the National AIDS Control Programme (now successfully in its fifth iteration) via government-run treatment centres with the goal of targeted intervention. Preventative measures such as pre-exposure prophylaxis, prevention of vertical transmission, and harm reduction are being focused on by countries in the region as part of a larger drive to meet the UNAIDS 95–95–95 target: 95% of people living with HIV should know their status, 95% of those diagnosed should receive ART, and 95% of those on ART should achieve viral suppression. Some regional exemplars stand out—for instance, Thailand, despite its high estimated prevalence and burden of HIV, became the first country in the region to achieve the elimination of mother-to-child transmission (EMTCT) in 2016. This success is attributed to their highly inclusive, integrated approach to care. Other countries in the region, such as Sri Lanka, Maldives, and Malaysia, have since followed suit to achieve EMTCT.

There are an estimated 3.5 million people living with HIV in the WHO South-East Asia region. According to WHO, only 85% are aware of their status, 74% are on regular ART, and 72% have reached viral suppression. This falls significantly short of the mark, indicating unaddressed needs in the region. New infections are concentrated in key populations—men who have sex with men, sex workers, transgender people, and people who inject drugs. Yet this reality is not uniformly reflected in policy. Evidence indicates that services intended for key populations are not funded proportionally in relation to their high vulnerability. For instance, India, Pakistan, Indonesia, and Thailand each direct less than 50% of HIV expenditure towards key populations. In addition to underfunding, key populations face persistent barriers including discrimination, stigma, and the risk of criminalisation often accompanied by severe legal consequences. These circumstances not only discourage individuals from accessing care but also foster risk-taking, which undermines public health efforts.

The impact of such barriers is evident, as illustrated by Rahman and colleagues in their study from Bangladesh featured in this issue. Among key population members in Bangladesh, in whom first-line ART was not effective, nearly one in five had virological failure and a quarter had a high viral load. Analysis of samples revealed mutations associated with resistance to first-line drugs, including lamivudine and efavirenz, indicating the emergence of drug-resistant strains. The potential causes of drug resistance need to be explored as they can serve as a proxy for understanding the systemic failures taking place. Without robust viral load monitoring, easy availability of second-line treatment, and drug resistance surveillance, we stand to lose hard-won ground.

These shortcomings in HIV response underscore a crucial point: for further progress to be made in the region, future efforts need to integrate social and legal protections into policy. Key populations should be enabled to seek and adhere to treatment in circumstances which allow them to be who they are without penalty. A significant milestone was reached on Jan 23, 2025, when Thailand became the first country in southeast Asia to legalise same-sex marriage. This decision conferred equal rights on matters of inheritance, adoption, and medical decision making—while the growing trend in rest of the world is towards withdrawing legal protection for vulnerable populations and cutting public health spending. Against this backdrop, the recent global funding crisis highlighted the need to shore up domestic resources as the majority of countries in the region are still heavily reliant on external funding for HIV response. The lesson for our region is clear: the way forward involves not only commitment to scientific advancement but also to social progress and financial backing.

Looking to the future, it is important to reflect on achievements and recognise the distance to be covered. The strategy for the region must be nuanced, vigilant, and multifaceted, with a focus on strengthening the logistics and surveillance of our treatment and prevention programmes. Community-based programmes are essential to bring services and outreach to where they are most needed. Prioritising the social and legal protection of people living with HIV is key to tackling the social drivers of the epidemic. As we stand at a crossroads between progress and complacency, we must remember that next 40 years will be determined by the choices made today.

